# Optic Nerve Sheath Diameter Correlation with Elevated Intracranial Pressure Determined via Ultrasound

**DOI:** 10.7759/cureus.4145

**Published:** 2019-02-27

**Authors:** Kamran Munawar, Muhammad Tariq Khan, Syed Waqar Hussain, Aayesha Qadeer, Zahid Siddique Shad, Sheher Bano, Azmat Abdullah

**Affiliations:** 1 Internal Medicine, Shifa International Hospital, Islamabad, PAK; 2 Internal Medicine, Khan Research Laboratories Hospital, Islamabad, PAK

**Keywords:** onsd, icu, tbi, icp

## Abstract

Background

The early detection of elevated intracranial pressure (ICP) can not only prevent mortality but also aid in more aggressive management. Brain computed tomography (CT) is a mainstay modality in detecting elevated ICP, but the feasibility of using brain CTs to detect elevated ICP in critically ill patients is limited, especially for patients who require high levels of inotropic support. The optic nerve sheath is a direct extension of the brain meninges. Therefore, the elevation of ICP is directly transmitted to the sheath. Measuring the optic nerve sheath diameter (ONSD) through ultrasound (US) is a bedside, noninvasive means to detect elevated ICP. The goal of this study was to assess the correlation of ONSD with elevated ICP as measured via US in an intensive care unit (ICU).

Methods

We conducted a six-month prospective, single-center, observational study of mass effect stroke patients aged 18 to 65 years who had a traumatic brain injury (TBI) and were admitted to the ICU. Patients with chronic hydrocephalus, extensive local orbit trauma, a pre-existing ocular disease affecting the optic nerve and/or orbital cavity, hyperthyroidism with exophthalmos, and facial trauma affecting the orbits and/or eyeballs were excluded. We measured the ONSD at the entry of optic nerve into the globe using two-dimensional (2D) US.

Results

One hundred patients were included in the study. Forty-nine patients had diffuse cerebral edema detected on CT scan correlating with increased ONSD notable via bedside US. The mean ONSD related to CT-detectable elevated ICP was 0.61 cm. The sensitivity for the ONSD cut-off value of ≥5.8 mm was 94% (95% confidence interval [CI], 84.05% to 98.79%), and the specificity was 96.08% (95% CI, 86.7% to 99.52%).The positive predictive value was 92.08% (95% CI, 86.28% to 98.96%), and the negative predictive value was 94.23% (95% CI, 84.47% to 98.00%).

Conclusion

The greatest accuracy in ONSD was found with a cut-off of >0.58 cm in patients with positive CT brain findings. Therefore, US can be used as an initial screening test when physicians suspect a patient has elevated ICP.

## Introduction

Elevated intracranial pressure (ICP) is a frequent problem in neurosurgical and neurological practice and may be due to inappropriate cerebrospinal fluid (CSF) circulation, intracranial mass lesions (including traumatic or spontaneous intracranial bleeding) or more diffuse intracranial pathological processes. Elevated ICP development may be acute or chronic, but it commonly results in a pressure gradient among compartments and a shift of brain structures causing changes in consciousness, bilateral ptosis, pupillary dilatation, and impaired up gaze. Elevated ICP can be assessed directly through the invasive measurement of intraventricular pressure [1]. However, elevated ICP can also be inferred by observing secondary effects associated with high ICP such as papilledema or dilatation of the cerebral ventricles. Lately, ocular ultrasound (US) for detecting dilatation of the optic nerve sheath diameter (ONSD) as a sign of elevated ICP has shown promising results. The optic nerve sheath is composed of dura mater and covers the optic nerve up to the back of the eye. Intracranial CSF is in direct connection with the rim of the subarachnoid space between the optic nerve sheath and the nerve itself. Therefore, any change in ICP will dynamically change the ONSD. When patients develop elevated ICP, ophthalmologists are usually consulted to assess the ONSD, as a clinical examination may be inadequate. Computed tomography (CT) and magnetic resonance imaging (MRI) are useful tools for detecting elevated ICP noninvasively. However, CT and MRI are not always indicated, especially in critically ill patients who require high levels of inotropic support or ventilator support. The use of bedside US has shown to be an equivalent if not improved means of measuring elevated ICP via assessment of the ONSD [2]. The aim of this study was to investigate the usefulness of using bedside US to measure OSND to determine if a patient has elevated ICP.

## Materials and methods

We conducted a six-month prospective, single-center, observational study in the intensive care unit (ICU) at Shifa International Hospital in Pakistan. The study included patients aged 18 to 65 years admitted to the ICU for stroke with mass effect causing traumatic brain injury (TBI). Patients with chronic hydrocephalus, extensive local orbit trauma, a pre-existing ocular disease affecting the optic nerve and/or orbital cavity and hyperthyroidism with exophthalmia, and facial trauma affecting the orbits and/or eyeballs were excluded from the study. The ONSD US findings were evaluated by trained fellows of critical care, and brain CT scan findings were masked to the US evaluators. The ONSD was measured via two-dimensional (2D) US scans at the entry of optic nerve into the globe. After sonography, a brain CT scan was conducted and reviewed by an expert radiologist and was compared with ONSD.

The hospital ethics committee approved the study protocol, and since the study was non-interventional, we did not require informed consent.

We followed a similar data collection to that described by Legrand et al. [3]. We collected patient age, Glasgow Coma Scale (GCS) score, and injury at the time of admission. We recorded the presence of signs showing elevated ICP on brain CT scan including massive intracerebral bleeding, subarachnoid hemorrhage with intraventricular extension, compression of the basal cistern, or midline shift >5 mm.

For an analytical description of quantitative variables, standard deviation and mean were used to assess the capacity for ONSD to predict brain CT results. We used a receiver operator characteristic (ROC) curve and its area under the curve to determine an optimal cut-off value for ONSD. Once the cut-off value was determined, we calculated the specificity, sensitivity, negative predictive value (NPV), positive predictive value (PPV), negative likelihood ratio, and positive likelihood ratio. P < 0.05 was considered statistically significant. We used IBM SPSS Statistics for Windows, version 21.0 (IBM Corp., Armonk, NY) in applying the ROC analysis.

All CT scans included in the study were performed in the radiology department at Shifa International Hospital. All patient surgical and medical histories were masked to the radiologist, as were the nature of the TBI and GCS scores at the time of assessment. CT scans were performed before any intervention when needed. Brain CT scans were performed with a series of slices. ONSD was measured vertically 0.3 cm behind the optic nerve entering the globe, and at this point, the transverse diameter was obtained separately on both eyes. A mean ONSD was produced based on the right and left eye measurements.

## Results

A total of 100 patients (53 men, 47 women) admitted to the ICU were evaluated in the study. The cutoff value for normal ONSD on 50 patients who had normal CT scans was 0.57 cm. Forty-nine patients with diffuse cerebral edema detected on CT scan also had increased ONSD on bedside ultrasonography. The average ONSD associated with elevated ICP detectable on CT was 0.61 cm. The sensitivity for the ONSD cut-off value of ≥5.8 mm was 94% (95% confidence interval [CI], 84.05% to 98.79%) and the specificity was 96.08% (95% CI, 86.7% to 99.52%); the PPV was 92.08% (95% CI, 86.28% to 98.96%), and the NPV was 94.23% (95% CI, 84.47% to 98.00%) (Table 1).

**Table 1:** Sensitivity and specificity of ultrasonography of optic nerve sheath diameter for diagnosis of elevated intracranial pressure

Positive if Greater Than or Equal To^a^	Sensitivity	Specificity
.00000	1.000	1.000
.39500	.959	1.000
.41500	.959	.980
.43000	.918	.980
.44500	.898	.922
.45500	.898	.902
.46500	.898	.863
.47500	.898	.765
.48500	.898	.667
.49500	.898	.529
.50500	.878	.431
.51500	.878	.314
.52500	.878	.255
.53500	.878	.176
.54500	.878	.118
.55500	.878	.059
.56500	.857	.020
.57500	.837	.020
.58500	.714	.020
.59500	.694	.020
.60500	.673	.020
.62000	.612	.020
.63500	.531	.020
.64500	.490	.020
.65500	.449	.020
.67000	.429	.020
.68500	.347	.020
.69500	.286	.000
.70500	.265	.000
.71500	.143	.000
.73000	.122	.000
.74500	.082	.000
.78000	.020	.000
1.00000	.000	.000
.0000	1.000	1.000
1.5000	.061	.980
3.0000	.000	.000

## Discussion

Elevated ICP can result in many clinical and traumatic circumstances and is a life-threatening condition [4]. The “gold standard” methods for measuring elevated ICP can put critically ill patients at further risk due to the invasive nature of the intervention, and these gold standard approaches carry a notable risk for severe complications like hemorrhage, infection, and malfunction. Therefore, these approaches are not indicated in patients in critical condition [5].

The assessment of ONSD for ICP detection has been widely studied via CT scan, MRI, and US [6]. The use of US for ONSD measurement is a recent, novel bedside approach to assess for elevated ICP. Bedside US is noninvasive and can provide a binary, satisfactory diagnostic accuracy [7]. However, the studies supporting this claim were limited by various methodological flaws to reach definitive results.

An ONSD of 5.8 mm as a cut-off value provided acceptable specificity, sensitivity, PPV, and NPV for detecting elevated ICP. The likelihood of elevated ICP is adequately low when the ONSD is less than 5.0 mm. A previous study reported the ONSD measurement cut-off value of 5.9 mm as an indicator of elevated ICP [8]. The lower PPV in that study, regardless of its higher specificity and sensitivity, could have been due to the smaller patient population with positive brain CT findings.

In the last few decades, numerous studies have revealed that ONSD can noninvasively reflect elevated ICP [9]. One such study even reported that an increase in ONSD occurs faster in the presence of elevated ICP than the signs visible via ophthalmoscope [10]. Girisgin et al. reported that the average ONSD in those patients with suspected elevated ICP was considerably higher than the average ONSD in healthy subjects [11]. Soldatos et al. reported that an ONSD >5.7 mm measured non-invasively to determine ICP had 74% sensitivity and 100% specificity compared to results found via invasive intraparenchymal catheter [12]. Major et al. showed a correlation of brain CT scans with ONSD >5 mm with a sensitivity of 86% and specificity of 100% [13]. A study of 156 children concluded that larger mean ONSD is associated with elevated ICP [14].

Best cut-off values for ONSD correlating to elevated ICP should be established and standardized for diverse subgroups categorized by age, gender, and condition. Optic nerve sonology use is limited due to its technical nature requiring specific expertise [12]. Sonography may not be possible in special situations such as patients with local area surgical wounds and or anatomical alterations due to trauma (e.g., head and facial trauma) [15].

Future studies shall be conducted in this regard to develop new and improved methods of ultrasonography in these critical situations, which provide an early, valuable and equivalent information if not above than CT scan brain.

## Conclusions

The greatest accuracy of US screening of ONSD for suspected elevated ICP is with an ONSD of more than 0.58 cm. Therefore, US ONSD screening can be a useful initial test for suspected elevated ICP, especially in patients for whom traditional CT scanning is not feasible.
